# Roboethics principles and policies in Europe and North America

**DOI:** 10.1007/s42452-021-04853-5

**Published:** 2021-11-07

**Authors:** Sofya Langman, Nicole Capicotto, Yaser Maddahi, Kourosh Zareinia

**Affiliations:** 1 Faculty of Medicine, University of British Columbia, Vancouver, BC, Canada; 2 Department of Biomedical Engineering, Faculty of Engineering & Architectural Science, Ryerson University, Toronto, ON Canada; 3 Department of Research and Development, Tactile Robotics, Winnipeg, MB Canada; 4 Department of Mechanical and Industrial Engineering, Faculty of Engineering & Architectural Science, Ryerson University, Toronto, ON Canada

**Keywords:** Roboethics, Ethics, Robotics, Artificial intelligence, Governance

## Abstract

Robotics and artificial intelligence (AI) are revolutionizing all spheres of human life. From industrial processes to graphic design, the implementation of automated intelligent systems is changing how industries work. The spread of robots and AI systems has triggered academic institutions to closely examine how these technologies may affect the humanity—this is how the fields of roboethics and AI ethics have been born. The identification of ethical issues for robotics and AI and creation of ethical frameworks were the first steps to creating a regulatory environment for these technologies. In this paper, we focus on regulatory efforts in Europe and North America to create enforceable regulation for AI and robotics. We describe and compare ethical principles, policies, and regulations that have been proposed by government organizations for the design and use of robots and AI. We also discuss proposed international regulation for robotics and AI. This paper tries to highlight the need for a comprehensive, enforceable, and agile policy to ethically regulate technology today and in the future. Through reviewing existing policies, we conclude that the European Unition currently leads the way in defining roboethics and AI ethical principles and implementing them into policy. Our findings suggest that governments in Europe and North America are aware of the ethical risks that robotics and AI pose, and are engaged in policymaking to create regulatory policies for these new technologies.

## Introduction

Robotics and artificial intelligence (AI) are having a profound impact on all aspects of everyday life: our food is collected by robots, we are being driven by self-driving vehicles, our phones know what we want to text to our loved ones, and when we get sick, our physician might be a robot. However, as exciting as these technologies are, they come with significant risks for the future of humanity. Over the last 10 years, the number of industrial robots has risen 300% and continues to increase [1]. In the next 10 years, as many as 20 million jobs worldwide may be displaced by industrial robots [2]. Automated systems are replacing not only manual laborers, they are replacing care workers, teachers, lovers, medical professionals, and soldiers. It is highly likely that in the future robots will co-exist with humans and contribute to society both physically and intellectually. It is not just the future of jobs that worries the public, but also the possibilities for inequality, decline in social wellbeing, and non-consensual control from corporations and governments [3, 4]. Due to these issues and the overall rise in robotics and AI research and implementation over the past decade, the development of ethical principles and regulation has become a priority for governments and organizations around the world.

The first attempts to understand the ethics of AI and robotics have come from academic institutions and private corporations, which demonstrates the field’s awareness of its potential implications. The study of the ethics of robotics, or roboethics, was pioneered by by Gianmarco Veruggio in the early 2000s [5]. Since then, roboethics and AI ethics have become widely discussed topics, with the number of publications mentioning either of the terms increasing tenfold in the last 5 years [6]. Although many organizations already propose well-considered ethical principles for robotics and AI, the need remains for enforceable ethical regulations on governmental and international levels. The need for regulation is felt by all members of the robotics and AI communities, which is why many non-government organizations have decided to create their own ethical policies independent of law and policy. However, this does not promote standardization of ethics, and allows for moral loopholes which could lead to creation of automated systems that infringe on human rights. Governments have recognized the potential and risks that AI and robotics bring, and have initiated the process for creation of legislation that accounts for ethical concerns in AI and robotics.

The goal of this paper is to summarize ethical frameworks and regulations in Europe and North America with specific focus on how ethical principles have been translated into law by government bodies in the European Union (EU), United States of America (U.S.), and Canada. This paper is structured as follows: we first provide a definition for robots and discuss how theoretical Laws of Robotics have initiated the discussion for ethical robots; we then move on to describe key ethical issues for robots; the following section describes ethical frameworks and regulations for Europe, North America, and as set out by international organizations such as the United Nations; lastly, we provide a brief discussion where we compare the progress made by Europe and North America in creating regulatory policies for robotics and AI, and list action-steps for establishing a regulatory environment that promotes ethical robotics and AI.

## Foundational principles of robotics and roboethics

While AI-powered robots are a thing of the present, there are examples of miraculous—for the time— machines from ancient civilizations which we can consider the first examples of robots. Al-Jazari, an Arab Muslim scholar from the thirteenth century BC designed wondrous items such as a programmable system for pouring and serving various drinks, a set of robotic musicians, and several water-raising machines [7, 8]. Leonardo da Vinci has also created a humanoid-looking automaton in a shape of a knight that was able move its head and jaw, wave its arms, and sit up [9]. The Industrial Revolution populated new technologies around the world and has permanently changed how people approach manual labor. With the discoveries of electricity, computers, and the internet, inventors were able to create machines and automatons capable of automating processes that were previously performed by humans. A new revolution is currently underway in the workforce, and over 70% of US workers indicate that they are worried about a future when robots and computers can perform human jobs [3]. Similarly, in a study that surveyed over 20,000 EU workers, the majority has indicated that they agree that robots steal people’s jobs [10]. As the capabilities of machines have changed, our definitions for robots and.

### Definition of robots

The term “robot” was first introduced by Karel Capek in a play that first premiered in Prague in 1921 [11]. The term, derived from Czech word “rabota” meaning compulsory work, was used to identify artificial laborers that served humans in a fictional Utopian society [11, 12]. Since then, the word “robot” has been popularized through works of science fiction and is now also applied to an array of intelligent mechanical systems. When considering policy design and implementation, it is critical to have a most complete and accurate definition of a robot, since a policy may or may not be applied to an object depending on whether it is classified as a robot. One the most widely accepted definitions for robot is one from the Robot Institute of America: “A robot is a reprogrammable, multifunctional manipulator designed to move material, parts, tools, or specialized devices, through variable programmed motions for the performance of a variety of tasks” [13]. Since the development of AI and the Internet of Things, and the merging of programmable systems with physical operators, the distinction between AI and robots is become more arbitrary [14]. As such, perhaps the most straightforward definition for robot is “an embodied AI” [15].

With the above definitions in mind, a robot should exhibit these three properties [14]:

- Programmability, or an ability for a designer to manipulate robot’s functions and capacities;
- Mechanical Capability, enabling a robot to act on its surroundings; and
- Flexibility, allowing the robot to operate in a variety of ways and adapt to different scenarios.

With the emergence of specific robot types, such as social robots, the qualities above may be expanded. This should be considered when attempting to create a policy that should only be applied to a specific type of a robot. While universal policies would be useful in establishing a basic governance of robots, it would be difficult to apply the same policy to a cargo-stacking robot, a self-driving car, and a robotic soldier.

### Asimov’s laws of robotics

The term robotics, referring to a branch of engineering that studies robots, was first used by Isaac Asimov in his novel “Runaround” [16, 17]. In the same novel, Asimov has introduced the first set of laws that dictate a robot’s behavior [16]. These laws lay the foundation for roboethics and established the first set of boundaries between humans and robots. The Laws of Robotics (Laws) discuss concepts of safety, obedience, and self-preservation:

1. A robot may not injure a human being under any conditions—and, as a corollary, must not permit a human being to be injured because of inaction on [the robot’s] part.
2. A robot must follow all orders given by qualified human beings as long as they do not conflict with First Law.
3. A robot must protect [its] own existence, as long as that does not conflict with the First and Second Law.

Asimov later recognized that the First Law did not extend to the human society overall and added an additional Zeroth Law that would supersede the First Law [18]:

0. A robot may not injure humanity, or, through inaction, allow humanity to come to harm.

While these laws were relevant when written in 1942 and 1985 (Clarke, 1993), they are not applicable to robotics today. The Laws employ abstract concepts and require robots to make moral judgements based on difficult-to-calculate probabilities [14]. When Asimov wrote his robots, he was not operating under constraints of reality and technological capability; he could take creative freedoms to smooth over any inconsistencies. To apply these Laws to the current state of robotics, they need to be revised with more specificity. However, when AI reaches the point of singularity, some of Asimov’s original Laws may become relevant again.

### Revisions to the Asimov’s laws of robotics

When Asimov proposed his Laws of Robotics, he could not envision the technological developments and the geopolitical climate of the twenty-first century. Building on the foundational principles of the Laws, several versions of the new Laws of Robotics have been proposed. According to Murphy and Woods, the main issues with the Laws are that they 1) assume that robots are solely responsible for human safety, 2) fail to explain how robots should interpret orders given by humans, and 3) ignore that many robots lack a self-protective component of autonomy [19]. As a result, the Three Laws of Responsible Robotics focusing on accountability, responsiveness, and control, have been proposed [19]:

1. A human may not deploy a robot without the human–robot work system meeting the highest legal and professional standards of safety and ethics.
2. A robot must respond to humans as appropriate for their roles.
3. A robot must be endowed with sufficient situated autonomy to protect its own existence as long as such protection provides smooth transfer of control to other agents consistent with the first and second laws.

These updated laws recognize that as robots are created by humans, the responsibility for robot actions lies on humans too. Assignment of responsibility is essential when creating legislation and policy, as policy enforcement requires accountability. In addition to that, the Laws of Responsible Robotics recognize that robots are a part of dynamic relationships that are built through human–robot interactions [19]. These new Laws are not exhaustive nor specific, but they provide a more realistic starting point for ethicists and policy makers.

Since 2009 industries have revolutionized their work by employing cloud computing, big data, and cyber-physical systems [20]. The robot industry has reached the point where robots are able to provide an attractive return on investment for replacing manual labor with machines [21]. Some predictions suggest that the number of industrial robot installations will increase 300% in the next 10 years, and in some industries over 40% of manufacturing tasks will become automated [21]. These figures suggest that human–robot co-working is inevitable and will be the next step in industrial evolution. The changes in global industry require a new set of ethical robotics rules, which would protect human workers from capitalistic drivers and guarantee a peaceful coexistence of humans and robots in the same workplace. New technological developments have also spread to the military and medical sectors, where robots are able to perform such disparate functions as destroying terrorist cells and caring for the elderly. Increases in robotic and AI capabilities has inspired several organizations to develop new robotic principles and manifests [22]. Additionally, the New Laws of Robotics (New Laws) have been proposed in 2020, which take into account morals of human actors [23]:

1. Robotic systems and AI should complement professionals, not replace them;
2. Robotic systems and AI should not counterfeit humanity;
3. Robotic systems and AI should not intensify zero- sum arms races;
4. Robotic systems and AI must always indicate the identity of their creator(s), controller(s), and own er(s).

These New Laws, while not specific, reflect on the current trends in implementation of robots around the world. They take into account the most troubling trends in robotics today, and place hard restrictions on them. The New Laws, however, do not include restrictive language, and cannot be interpreted as a piece of regulatory policy. Given changes already observed in the field, it is likely that there will be other laws proposed in the future. This reflects on the iterative process in both technological developments and policymaking.

## Ethical questions for robotics policies

The development and marketization of robotics pose many ethical questions for researchers, practitioners, government, and society alike. These ethical questions lay a foundation for ethical principles, which in their turn facilitate creation of roboethical standards such as BS 8611 [24]. The combination of ethical frameoworks and standards informs creation of regulatory policies for robotics. In order to create relevant policy, governments and corporations have proposed various means of evaluating a robot. Here we discuss three categories based on which a robot can be evaluated to create ethical policy.

### Functionality

Robots are designed to perform various functions, from assembly of heavy machinery to patient care. Most commonly robots are designed to perform tasks with utilitarian purpose, where a robot performs repetitive or heavy tasks in a workplace [17]. These robots are often referred to as industrial robots, and they have been responsible for revolutionizing production economies around the world [17, 20]. Robots with more sophisticated mechanical functions were then adapted in medical fields, so we have seen the appearance of medical robots. Rapid advances in AI technology have facilitated development of a whole new class of robots- social robots. Social robots possess an ability to interact with humans, enabling them to perform caregiving, teaching, and customer service functions [25–27]. Outside of professional environment, robots are designed as toys, art objects, or exclusively for user pleasure [28–30]. Robots can now be expected to be involved in all aspects of human life, which undoubtedly will shape society and economy.

Robot’s intended functions can pose an array of ethical concerns. While the existence of industrial robots is somewhat accepted, there are questions being raised about how industrial robots will impact the workforce dynamics and the state of the world economy. Many workers are concerned about losing their jobs to robots, and industries are expecting that they will have to retrain their workers due to robot integration [3, 31, 32]. Some countries would be able to survive this industrial revolution, but for developing countries and regions relying on manual labor this change may prove to be disastrous [33]. Social robots present some of the similar challenges in relation to workforce dynamics, but also pose additional questions regarding dehumanization. Expansion of aging population has already caused Japan to look into robotic care for the elderly, and North America and Europe are likely to follow suit [34, 35]. Robotic caregivers may be able to assist with physical side of patient care, but it has been widely acknowledged that human contact is just as important to care and therapy. Care robots are not able to empathize with their charges and could exacerbate the problem that they were designed to solve by perpetuating loneliness and eroding their patient’s sense of dignity [35, 36]. Similar concerns can be raised in relation to robot childcare and education, where children may find themselves attached to robots in a way that would impact their social development [37, 38]. It is plausible that in the future robots would be able to perform any function a human can, so current policymakers could start questioning which roles robots should play in human society.

A robot may also have functions that are complimentary to a robot’s main function. For example, a robot designed to entertain the elderly may also be collecting user data such as sound, video, etc. Social robots can access user’s physical spaces and gain insights on user’s innermost thoughts, which could be of special concerns if robots are interacting with vulnerable populations [39]. In this scenario, data handling and ownership pose additional ethical concerns which would need to be addressed through policy. If robots have internet connectivity and upload data to servers, data security would add another layer to the potential regulatory framework.

There is another class of robots whose function is widely debated on an international scale, and which deserve a special mention here: military robots. At least 50 countries have either bought robots for military purposes or have military robotics programs; the U.S. Department of Defense has allocated $7.5 billion to development of unmanned systems in the fiscal year 2021 [40, 41]. Militaries around the world have been using robots to conduct reconnaissance, do surveillance, disarm landmines, and engage with targets [42]. It is the latter kind that is of outmost concern for the ethicists. Lethal autonomous weapon systems (LAWS) offer several advantages for the military: they have a potential to reduce casualties, improve precision, and provide continuous surveillance and analysis of the battlefield [43]. To understand ethical concerns for LAWS, we should ask three questions: 1) What can they do? 2) What should they do? and 3) What is their role in global security and likelihood of future wars? [44] The concern for human dignity is central to ethics of LAWS: they could eliminate the human judgement component from lethal confrontations, thus shifting the ethical burden for taking a life from humans to a machine [44, 45]. Whether LAWS should be able to make life and death decisions and follow up with violence based on that decision is the cornerstone question for ethicists and policymakers. Some researchers argue that LAWS should be under meaningful human control even after deployment, thus limiting the autonomy of such robots and still permitting for human-directed decision-making [42]. This point of view is supported by governmental organizations, for example the European Parliament resolution on a comprehensive European industrial policy on AI and robotics notes that “automated weapons systems should continue to have a human-in-command approach to artificial intelligence” [46]. A survey from 2015 has indicated that the majority of people opposes use of LAWS for offensive purposes and supports international ban of LAWS [47]. If International Humanitarian Law does allow the use of LAWS, it would be the up to robot engineers to create algorithms that determine the extent of human involvement for ethical robotic warfare [48].

### Capability

While two robots might be designed to perform the same function, their capabilities may vary depending on a unit’s hardware and software. In other words, a robot that is only capable of simple processing operations cannot be compared to a robot that has sophisticated AI with a capacity for learning. The more advanced the AI, the more ethical questions can be raised to create regulatory policy. With AI that possesses human-like capabilities, one might even start asking whether such sophisticated robots deserve to have rights that would traditionally be granted to intelligent life forms. Further, if a robot starts to develop capacity for independent thought, would it be ethical for humans to regulate it as an object or a property? These questions could be further complicated by the anthropomorphization of robots, which would create an illusion of social bonding between a robot and a human [49–51]. Unlike the future concerns for human-like AI, social robots already present anthropomorphization concerns for the ethicists today. Humanization of robots can result in more positive attitudes towards robots, but also creates an unrealistic perception of robot capabilities [52]. It would be up to policymakers to weigh the pros and cons of robot humanization and decide whether regulation of robot design and social programming should be implemented.

If AI evolves to the point of human intelligence, it is also possible that robots will develop a capability of deception [53]. Ethics of deception in human behavior are widely debated, so incorporation of deception for robots presents a similar ethical challenge. The most common view of deception is that it is wrong to mislead others, especially for personal gain, but it might be acceptable to lie for a “greater good” [54]. If robots were to follow that rule of thumb, roboticists would have to define situations in which deception would be acceptable. In this case, the same moral and ethical standards extend from humans to robots. There are, however, some robot-specific forms of deception that cause special concern for robotics researchers. Dishonest anthropomorphism refers to robots using deceptive signals to conceal a capacity it possesses, or to suggest that is has a capacity that it does not possess [55]. For example, a social robot could overt it’s “gaze” pretending that it does not see a human when in fact it is recording via a hidden camera [56]. Regulatory policies would need to take into account robots’ deception ability and formulate rules for public awareness.

### Autonomy

Autonomy refers to a robot’s ability to perform operations and adapt to changes independently from humans. Balancing robot autonomy and human control is one of the core challenges in robotics from both ethical and technical perspectives [57]. This challenge is applicable for all types of robots, and the expectation is that robots should behave autonomously while performing both technical and social tasks [57]. Robots can be assessed based on the amount of autonomy they possess. Several scales have been created to assess autonomy levels in different kinds of robots. For example, Attanasio et. al. have ranked autonomy of surgical robots from 0 to 5, where 0 refers to robotic systems fully operated by the human surgeon, and 5 referring to systems that can perform surgery with no human input [58]. There are currently no surgical robots operating at Level 5 of Autonomy, but there are systems that operate at Level 4. This type of a surgical robot can interpret operative information, devise an action plan, adjust, and execute the plan while operating autonomously under surgeon’s supervision [58, 59]. Higher levels of autonomy also bring up questions about moral responsibility and accountability. If a robot has an ability to make decisions, would the robot also be responsible for the consequences? This is an especially important question in the context of policy and legislation. There are currently several cases in court where Tesla self-driving cars were involved in accidents where people died [60, 61]. These lawsuits might be critical in establishing precedent for accountability policies in autonomous robotics.

Robot’s level of autonomy influences how much humans trust robots. This is especially the case for robots that operate in human-rich environments, such as social robots and medical robots. A study surveying public’s opinion on surgical robots found that 69% of responders felt very uncomfortable about robots performing surgery without direct control from a surgeon [62]. Another study found that non-physicians were more likely to choose robotic surgery compared to physicians [63]. Since we can expect robots and humans to co-exist and collaborate, fostering trust for autonomous robots would be fundamental to their ability to perform their tasks. Policymakers will need to be cognizant of public’s perspectives and implement effective strategies in launching human–robot co-working arrangements [64].

The above sections suggest certain scenarios, factors, and concerns to consider surrounding the use of robotics in society. Implementation of robotics will have far-reaching effects on the future society. Diversity in robot functions, capabilities, and autonomy complicates the creation of a unified set of rules. However, the use of robotics may pose risks to different groups of individuals, and it is essential that the appropriate stakeholder is held responsible in harmful situations. Ultimately, the extent of ethical concern is informed by the human–robot interactions a particular robot can produce. Criteria like robot functionality, capability, contact with humans, and requirement for social skills may be taken into consideration in evaluating the ethics of human–robot interactions, and by extension the robot itself [65]. Public’s safety depends on policy and legislation, so it is critical that policymakers recognize ethical implications of individual human–robot interactions and effects robots have on society overall when creating policies to govern robotics. It should also be noted that we as humans create, use, and govern robots, which means that roboethics and robot regulation depend on ethical humans to create and uphold these frameworks.

## Roboethics and AI policies in North America and Europe

Creation of the regulatory framework for robotics is a lengthy multi-step process. Identification and distribution of ethical principles is the first step in creating relevant policy to govern the robotics field. A variety of organizations around the world has come up with their version of principles for ethical robotics. Governments and global organizations take these principles under advisement to create policies, thus translating best-practice suggestions into legislation. However, ethical guidelines often stay on as principles, thus creating an attractive loophole for companies to avoid regulation and legal persecution [66]. In cases where institutions integrate ethics into their mandate, it serves only as a marketing strategy and not a binding agreement [67]. One of the biggest gaps in AI and robotics ethical frameworks is the strict regulatory aspect. The next section examines the state of roboethics regulatory policies as proposed by international governing organizations, and North American and European governments. The policies discussed include executive orders, resolutions, proposals, and official reports commissioned by governmental agencies. Further, we have decided to include both AI and robotics policies in this overview. There are significant similarities in the ethics of robotics and AI, especially if we view robots as embodied AI [6]. The governments have also dedicated more attention to regulation of AI, most likely because AI technology is being actively incorporated in all aspects of life. It is also possible that as technology develops the line between robotics and AI will blur to the point that the distinction will be not important for the purposes of law [68]. It should be also noted that robotics technology is advancing faster than regulatory policies, so laws approved today may need to be changed in the future to reflect the state of the field of robotics [69, 70]. The latter concern also reflects on the changing and philosophical nature of ethics, where creating long lasting laws might be a challenge.

### European roboethics principles, policies and legislation

European policies for robotics are initiated by the European Parliament, the European Commission and by individual governments in the EU (also referred to as Member States). Given the trade and research partnerships that exist between European countries, legislation set out by the European Parliament and the European Commission has the most power to direct the field of robotics in Europe. Following this, the review focuses on the roboethics and AI principles set up by the European Parliament and Commission. Detailed descriptions for robotics and AI policies from individual countries can be found elsewhere [71, 72].

#### European ethical framework for robotics

A study commissioned by the European Parliament Legal Affairs Committee on European civil law in robotics proposed a general ethical framework to be followed in future legislation by the Parliament. The framework focuses on roboethical principles that would protect humans from robots and covers concepts of safety, liberty, privacy, deception, and equality [73]. The 2017 resolution of the European Parliament on the civil law rules on robotics and AI prioritized six main areas for EU legislative efforts: ethics, liability, intellectual property and flow of data, standardization, employment and institutional coordination and oversight [74, 75]. Additionally, the 2017 resolution included recommendations for a code of conduct for robotics scientists, where the role of ethical design and responsible research was recognized [75].

In a later statement published in 2018, the European Group on Ethics in Science and New Technologies listed roboethics principles that align with the current EU Treaties and the EU Charter of Fundamental Rights [76]. These principles are summarized below:

- *Human dignity* Autonomous technologies must not violate the inherent human right to be respected.
- *Autonomy* Humans are free to live to by their own standards, and humans are responsible to exert control over autonomous technologies. Autonomous technologies must not impair human freedom, responsibility, and control.
- *Responsibility* The development and use of autonomous technologies must benefit society and the environment on a global scale. Such benefits must be defined by democratic means.
- *Justice, equity, and solidarity* Regulators and practitioners must prevent or neutralize discriminatory datasets from training AI systems. AI should further efforts in global justice and equality. All humans should benefit from autonomous technologies.
- *Democracy* The regulation of autonomous technologies must result from democratic, public debate, and engagement.
- *Rule of law and accountability* Regulation of autonomous technologies must uphold all human rights standards, such as protections for safety and privacy. These protections rely on rule of law, access to justice, the right to redress, and the right to a fair trial.
- *Security, safety, and bodily and mental integrity* Safe autonomous systems promote external, internal, and emotional safety. External safety protects environments and users. Internal safety ensures consistent performance and protects against hacking. Emotional safety protects users from exploitation and abuse when interacting with autonomous machines.
- *Data protection and privacy* Digital communication technologies employ autonomous technologies to amass and store vast quantities of users’ personal data. Therefore, autonomous technologies challenge protections on personal information and privacy.
- *Sustainability* Autonomous technologies must align with our human responsibility to protect our planet’s ability to support life, to preserve the continued quality of the environment, and to maintain the prosperity of our species.

The roboethics principles, as defined by the European Commission in 2018, are further supplemented by the resolution on the European industrial policy on AI and robotics that was published in 2019 by the European Parliament [46]. The resolution recognizes the role of ethics in robotics and AI regulation and specifically focuses on four aspects of the roboethical framework: 1) human-centric technology; 2) embedded values in technology; 3) decision-making, and 4) transparency, bias and explainability of algorithms [46].

European commitment to ethical AI and robotics has since been re-confirmed by Ursula von der Leyen, the President-Elect of the European Commission. Her agenda for Europe specifically includes creation of standards for new generation of technologies and legislation for coordinated action on AI [77].

#### European Policies for Ethical Robotics and AI

European policy may be viewed from both national and international perspectives. Countries in the EU are free to set their own rules, but are also required to follow policy set out by the European Parliament and European Commission. In fact, current opinion of the European Union is that in term of ethical regulation for robotics and AI, there is a need for coordinated action between EU member states and the European Commission [74]. In order to create relevant policy, the EU has conducted thorough studies that informed the policymakers on current perspectives of stakeholders. This section summarizes these efforts and lays out key aspects of existing robotics regulatory framework in Europe.

##### Robolaw project

In 2012 the EU initiated a collaborative project RoboLaw to investigate how emerging technologies will challenge European legal systems and to survey the state of the existing robotics regulation [78]. The project produced a report with recommendations for the European Commission on regulating robotics and related technologies [78, 79]. The report details two approaches to robotics legislation: 1) creation of new laws to accommodate the new technology and 2) adaptation of existing laws to reflect technology developments. In view of the scope of the robotics field, the authors argue that both approaches might need to be employed by policymakers [79]. To investigate this question further, RoboLaw authors chose a case-study approach where they profiled four specific robot types: self-driving cars, computer-integrated surgical systems, robotic prosthesis, and care robots [78]. This approach was especially effective in prioritizing human rights and identifying unique concerns that could be missed if a broader approach was employed. Further, while each type of a robot does present a unique legislative challenge, the authors were able to deduce common themes, such as liability, on which policymakers could act. Overall, the RoboLaw project has created a foundation for robotics regulation in the EU and promoted developments in the European legislature to accommodate robotics and AI regulation.

##### European regulatory framework for ethical robotics and AI

EU and its members already possess a rich legislative framework that is capable of accommodating some of the roboethical principles outlined above. However, both the European Parliament and the European Commission, highlight the need for further evaluation of emerging technologies and acknowledge that current legal framework is not sufficient to address all challenges posed by robotics and AI [75]. The SIENNA Project, an EU initiative aimed at understanding of ethical and human rights challenges posed by new technologies, generated a report that maps existing EU legislation to key legal issues in robotics and AI [80]. Issues of safety, liability, privacy, and equity are amongst the most well-defined by the current laws [80, 81]. Other ethical concerns for robotics, such as legal personhood for advanced AI systems, currently don’t have any existing legal framework. The latter is understandable given that AI has not reached that level of advance, but even for ethical issues that do have legal coverage, existing laws are not always specific to robotics. For example, product safety is extensively covered through Directive 2001/95/EC on general product safety and Regulation (EC) No 765/2008 on market surveillance [82, 83]. These regulations were written more than 10 years ago and do not reflect on developments in digital technologies: issues such as connectivity, autonomy, algorithmic opacity, and data dependency are not explicitly discussed in the current legal product safety framework [81]. An additional challenge is presented by the variety of new technologies, where certain types of robots will need to be covered under additional legal frameworks. For example, transportation robots could be regulated though regulations such as Regulation (EU) 2018/858 on approval and surveillance of motor vehicles [73, 84].

An alternative way to regulate AI was recently proposed by the European Commission: Proposal COM(2021)206 for harmonized rules on AI develops a legal framework based on risk-levels for AI systems [85]. This approach received positive feedback during an online public consultation with European stakeholders [86]. Higher assessed risk of an AI system would mean more stringent regulation from the EU and the member states. AI systems categorized as high risk included algorithms for education, law enforcement, and worker management [87]. A similar approach can be applied to robotics, especially in systems where AI and robotics converge into a single system. Evaluating a robot based on its functionality, capability, and autonomy can inform how much risk it poses to humans, and how it should be regulated. Risk-based regulation has several advantages over the traditional legal framework: 1) flexibility; 2) proportionality; 3) complementarity and consistency with the current legislation. It is the opinion of the European Commission that if risk-based framework is adopted by EU and the Member States, it will allow for innovation while ensuring respect for existing laws and values [85].

##### Ethical regulation of research and innovation

Research and innovation comprise one of the main focuses in the EU strategy for sustainable growth and prosperity. Responsible Research and Innovation (RRI) is a new governance model proposed by the EU that puts emphasis on co-creation and co-production with society [88, 89]. RRI framework has three main features:

1. Emphasis on science for society, where research efforts are focused on the “right impacts”;
2. Development of mechanisms for reflection and inclusion, where research goals are achieved ethically, inclusively, and democratically; and
3. Responsibility, where RRI framework is applicable not only for researchers, but also entrepreneurs, policymakers, funding organizations, etc. [90, 91].

When considering the implementation of the RRI, the European Commission identified four main options that ranged from a “business as usual” option (no further action required), to legally binding initiatives on that would promote RRI coordination between the EU member states [92]. To date, the EU has implemented several policies that reflect on RRI principles and launched numerous RRI projects via the Horizon 2020 program [93].

The Horizon 2020 program has funded €80 billion worth of projects, which include research initiatives, societal profiling for identification of unique challenges, and educational programs [93, 94]. One of the most powerful ways to regulate research is by controlling funding. To receive funding from the Horizon 2020, Digital Europe, or European Defense Fund programs, an applicant must complete an ethics self-assessment if their proposed research is in areas with high ethical risk [95]. The self-assessment helps applicants ensure that their research will comply with applicable international, EU and national law, and also facilitates grant review with regard to responsible implementation and social acceptance [95]. Importantly, completion of the self-assessment is a part of one’s grant agreement and imposes binding obligations that may be verified through official channels. For artificial intelligence, and by extension robotics, the assessment relies on the guidelines proposed by the High-Level Expert Group on AI [96, 97]. To be approved for funding, the project must follow key prerequisites for ethically sounds AI:

1. Human agency and oversight,
2. Technical robustness and safety,
3. Privacy and data governance,
4. Transparency,
5. Diversity, non-discrimination and fairness,
6. Societal and environmental wellbeing, and
7. Accountability [97].

Apart from the above prerequisites, grant applicants are invited to consider how their AI system may be used, and whether it can be utilized in especially dangerous manner, for example in weapon systems [95].

The EU has developed a robust multi-project framework to support their RRI efforts and conducted case-studies to understand best implementation strategies across EU Member States. One of the deliverables includes a handbook for organizations aimed at strengthening responsible research and innovation [98]. The handbook can be utilized by academic institutions to foster RRI among engineers-in-training, this creating professionals that would follow the EU directive for ethical by design robotic systems [99]. The diversity and expanse of RRI initiatives is likely to facilitate ethical implementation of robotics and AI in EU.

It is evident that Europe is currently actively implementing roboethics principles into policy. Europe’s approach to policymaking is characterized by thorough research, extensive survey of all stakeholders, and prioritization of common goals of growth, innovation, and sustainability. European governing bodies are aware of the weaknesses of the existing legislation and have created a comprehensive list of enhancements that would bring legal frameworks for robotics and AI to the next level [100]. Overall, the EU’s commitment to democratic policymaking fosters close relationships between industry, academia, and government, which creates a collaborative environment for relevant policymaking.

### North American policies for ethical robotics and AI

America has the least number of robot units sold or shipped as compared to Europe and Asia [101]. This difference can be explained by a variety of factors such as economic policy, cultural climate, funding for innovation, and regulatory framework. Unlike Europe, North America does not have a unified governance system, so this section will explore roboethics principles and policies in the United States of America and Canada. Both countries represent unique governance frameworks that significantly differ from the European legislative system. In both the U.S. and Canada, federal laws are supplemented by state and province legislation, thus complicating the creation of a unified regulatory framework for robotics. Additionally, the Canadian and American common law systems rely heavily on precedent setting case law, where regulation is created based on precedents; this is especially troubling for emerging technologies such as AI and robotics because ethical issues might not be immediately brought up in the court system. There are also cultural differences between Europe and North America- where the EU places a lot of emphasis on sustainable growth, stakeholder engagement, and democracy, the U.S. is heavily focused on productivity and economic growth [102]. As such, there is a paucity of government-set ethical frameworks for AI and robotics. Here we discuss the existing regulatory and ethical frameworks for robotics and AI in North America.

#### American ethical frameworks for robotics and AI

U.S. interest in AI and robotics leadership is evident: several executive orders, memorandums, reports, and strategies from the Office of the President outline U.S. national goals for AI development and leadership [102–105]. The National Science and Technology Council has published a strategic plan for AI research, where the Council recommends development of a framework that would support effective AI innovation and ethical implementation [106]. The U.S. Congress has also passed the National Artificial Intelligence Initiative Act of 2020 which established the National AI Initiative to help coordinate AI-related activities across U.S. Departments and Agencies [107]. On February 11, 2019 President Trump signed an executive order which presents five principles for the American AI Initiative. These principles reflect on several core themes:

1. Commitment to AI development and implementation to support economic competitiveness and national security;
2. Creation of technical standards for AI deployment and adaptation;
3. Education of the public to help people develop skills necessary in the future (when AI will become more prominent);
4. Fostering of public trust and confidence in AI technologies and protection of civil liberties, privacy, and American values;
5. Creation of international environment that would support American AI industries [104].

These principles reflect on core ethical values of safety and trust, but don’t provide enough detail to be read as regulatory policy. The majority of specific ethical principles and frameworks are released by individual departments, such as the Department of Defense (DOD), the Department of Transportation (DOT), and the Department of Health and Human Services (HHS) [108–110]. The former has published five qualities of ethical AI, which are aimed to supplement existing U.S. military ethical framework [108]. These qualities are as follows: responsible, equitable, traceable, reliable, and governable. DOT has taken a less structured approach but has also demonstrated commitment to ethical regulation in their report on the future of automated vehicles. The report lists key concerns that DOT has identified though stakeholder engagement, many of which overlap with ethical concerns for robotics and AI [109]. Overall, the U.S. currently does not have a unified government-presented ethical framework for AI and even less so for robotics.

In the current system, it is up to individual departments, institutions, and industries to evaluate ethics of their activities and devise an ethical framework to be followed. There are several notable examples where a non-governmental institution has created an ethical framework that later got widely adopted. The Future of Life Institute has created 23 Asilomar Principles which reflect on research, ethics and values, and long-term issues for AI [111]. The Asilomar Principles have gathered a large following in the field and have been signed by over 1500 AI/robotics researchers. Further, the California Office of Legislative Council has passed a resolution in support of the Asilomar Principles [112]. Another example comes from Partnership on AI, a consortium founded by Amazon, Facebook, Google, Microsoft, Apple, and other technology executives [113, 114]. They have created a list of tenets that promote research, discussion, education, understanding, and leadership in the development and use of AI technologies. Per one of the tenets, the Partnership’s members are supposed to “ensure that AI technology is robust, reliable, trustworthy, and operates within secure constraints” and “promote safeguards and technologies that do no harm” [114]. In contrast to this tenet, Apple is currently preparing to implement a non-voluntary AI algorithm to scan for images of child sexual abuse on iOS devices, thus implementing an AI with major risks and creating a precedent for privacy violation on a world-wide scale [115, 116]. This last example illustrates a major weakness of a privately set ethical framework that is not supported on a government level.

#### Canadian ethical frameworks for robotics and AI

Over the last decade the Canada has demonstrated an interest in becoming a global leader for AI. To reach this goal, the Canadian government has established several programs to support ethical AI development and deployment. In 2017 the Canadian government committed $125 million for a pan-Canadian Artificial Intelligence Strategy, the objective of which is to attract world-class AI talent, foster a collaborative AI ecosystem, and understand the societal implications of AI [117]. Since its launch, the AI Strategy has resulted in increased AI skills migration, a 200% increase in the number of publications from AI institutes, and a 49% boost in investor funding for AI projects [118, 119]. While the Canadian Institute for Advanced Research (CIFAR) was developing the AI strategy, they conducted stakeholder interviews, which included workshops and discussions with the Indigenous community. In collaboration with the Initiative for Indigenous Futures, CIFAR has released a position paper that discusses guidelines for Indigenous-centered AI design. Notably, the paper specifies that while these guidelines were developed with Indigenous community, they are applicable in any context where AI development and implementation is considered [120]. Some of the guidelines listed are similar to ones proposed in other ethical guidelines for AI and robotics (e.g. data management, ethical design, need for governance guidelines, responsibility and accountability), but there are some notable differences. Specifically, guidelines for locality, relationality and reciprocity, and recognition of the cultural nature present valuable additions to traditional ethical frameworks. Recognition of cultural differences and locality of AI applications will have significant influence of the future of robotics and AI once these technologies become globally widespread.

The Canadian government has recognized the opportunities that AI and digital technologies present for the future of governance. The Digital Government program lists five guiding principles to ensure effective and ethical use of AI [121]. Under these principles, the government will.

1. Understand and measure the impact of AI,
2. Be transparent about how and when AI is being used,
3. Provide explanations on AI decision making,
4. Be transparent about sharing technical details, and
5. Provide sufficient training on the use of AI solutions [121].

Further, the Government of Canada has launched an international collaboration with France to guide responsible adoption of AI worldwide through the International Panel on Artificial Intelligence (IPAI) [122]. The goal of IPAI is to conduct analysis to guide AI policy development grounded in human rights, which will be achieved through production of reports and assessments. This collaboration is a promising step to creation of universal ethical frameworks for AI.

Outside of governmental efforts to create ethical guidelines for AI and robotics, Canadian academic institutions and non-profits have made significant contributions to the field. The University of Montreal proposed the Montreal Declaration for Responsible Development of Artificial Intelligence, which has been signed by 192 organizations and 2300 citizens [123]. The Declaration lists 10 principles for ethical AI and provides recommendations for development of public policies on AI [124]. The Canadian Robotics and AI Ethical Design Lab (CRAiEDL), based from the University of Ottawa, has explored roboethics of healthcare, social robots, automated vehicles and weapons [125]. The works of CRAiEDL members have been published in academic journals and further propagated through stakeholder presentations. The group has also created ethical design tools for robotics and AI, which provide practical techniques to empower engineers to engage in the ethical-by-design process [126]. The Open Roboethics Institute (ORI), a nonprofit think tank from Vancouver, has also created an AI Ethics Assessment Toolkit that walks organizations and individuals though a step-by-step process for design of impactful technologies that are aligned with shared values of the society [127].

Compared to the U.S. ethical frameworks, the Canadian system is similarly lacking in terms of robust federal initiatives for ethical AI and robotics. A report published by the University of Montreal stresses that current interconnection between government, industry, and academia largely benefits private interests [128]. Currently private AI companies receive funding from the Canadian government while following internal ethics frameworks, and what is more worrisome is that companies that have been linked to human rights abuses are allowed to become vendors with the government [128–130]. Overall, the combined work of CIFAR, academic institutions, and non-profit think tanks creates a solid foundation for creation of much-needed governmental policies on robotics and AI.

#### American regulatory framework for ethical robotics and AI

Currently there is no single governmental body responsible for AI and Robotics regulation although creation of a Federal Robotics Commission has been proposed [131]. As a result, current policy and legislature on robotics and AI in the U.S. has been fragmented and generally falls under one of these categories: federal and state law, case law, and technical standards.

On the Constitutional level, there have not yet been any amendments to reflect on the impact robotics and AI have had. The Fourth Amendment protecting citizens from unreasonable search may be in the biggest need for revision due to incorporation of AI-directed risk-assessment tools and robotics in law enforcement [132–134]. If robots reach an independent status, the First Amendment would also need to be revised to include AI-generated speech [135]. The existing legislation is primarily focused on specific applications of robotics and AI such as unmanned aircrafts, self-driving vehicles, algorithms for data collection and assessment, and facial recognition technology [136]. From the roboethics perspective, most of these policies reflect on the principles of privacy, accountability, and transparency [137]. The U.S. Congress has a few bills awaiting enactment, such as the Algorithmic Accountability Act which would require companies to conduct risk-assessment on automated decision systems based on their “accuracy, fairness, bias, dis- crimination, privacy, and security” [138]. If passed, the bill will regulate AI across industries and will be enforced by the Federal Trade Commission [137, 138]. In addition to robot-specific laws, there are also laws that apply to robots by extension, such as regulations on product safety, and by extension manufacturer liability [139, 140]. There are also regulations for safe use of robots in the workplace: Occupational Safety and Health Administration has created its guidelines for robotics safety in 1987 [141]. These guidelines will need to be adapted to reflect technological development, societal changes, and current workforce expectations [142].

Wide implementation of AI and robotics in society has produced several legal precedents in the U.S. courts. Undoubtedly the number of cases involving AI and robots will continue to rise, so laws and court decisions made now will have long-lasting effects in the future of robotics case law. Ryan Calo has separated existing cases based on the role a robot plays in the trial— is the robot an object or a subject of the judiciary system [143]. If a robot is designated as an object, then is acts as an artifact in a human world, e.g. a surgical robot being manipulated by a surgeon results in an unfavorable patient outcome [143, 144]. In this type of case-law, liability assessment presents a major challenge. One of the first influential cases on AI and liability comes from 1949 case Brouse v. United States, where the court decided that the pilot has to keep a proper and constant lookout even if the plane is controlled via an autopilot [145]. The same case can be viewed from the autonomy perspective— in this case the autopilot was not capable of detecting sudden changes and making autonomous decisions to change flight direction. The courts have also tried cases pertaining to algorithm transparency: in State v Loomis, the court decided that use of a risk-assessment software for sentencing did not violate rights of the accused, even though the algorithm specifics were not disclosed [146, 147].

In Calo’s case law framework, if a robot is designated as a subject, it only appears in the imagination of the judge [143]. While not as applicable to robotics regulation, perception of robots shapes future rights of robots. Currently robots are largely viewed as tools, impartial and inanimate objects, incapable of independent action [143, 148, 149]. As a result, there have been cases where humans were compared to robots, and assigned robot-like characteristics. If the judicial system adjusts its perception of robots to more independent machines, the robot analogy would be unusable, which would alter the outcome for the cases in question. The perception of robots and AI by humans will also ultimately affect the rights of autonomous systems. It is conceivable that future AI will reach human-like capabilities, but even in the current day AI systems are capable of creation. A unique case was recently heard by the District Court of Virginia, where an AI engineer had sued the U.S. Patent Office for declining two patents that he had submitted on the behalf of the AI system, which he designated as an inventor on the patent documents [150, 151]. The court has sided with the U.S. Patent Office and ruled that AI cannot be an inventor [151]. This ruling will undoubtedly affect how the work of AI, especially in terms of ownership, will be perceived in future court cases. Overall, the court system may expect to face a difficult task of deciding on robotics cases in a rapidly changing technological and legislative environments.

Technical standards can be used by industries to ensure that all products meet a pre-set benchmark and thus provide another layer of regulation for AI and robotics. Institute of Electrical and Electronics Engineers was founded initially as a U.S. professional organization but has since grown internationally to include over 420,000 members across 160 countries [152]. IEEE has previously published guidelines on ethical AI development, and in 2016 IEEE pioneered a new set of technical standards that prioritizes ethical considerations in the design and manufacture of robotics and AI [153, 154]. The P7000 family of standards could be helpful in assisting lawmakers in designing policy that is ethical and supported by the industry [155, 156]. For example, P7001 “Transparency of Autonomous Systems” could supplement the recently introduced Algorithmic Justice and Online Platform Transparency Act and promote development of trustworthy and ethical AI [157, 158]. The Algorithmic Accountability Act could be accompanied by IEEE’s P7003 on “Algorithmic Bias Considerations” [159]. IEEE has also created a framework for assessing the impact of autonomous and intelligent systems on human well-being, which is one of the very few standards that address ethical design for robotic devices [160].These standards are informative in shaping government policy, and if adopted could facilitate establishment of ethical robotics worldwide.

#### Canadian regulatory framework for ethical robotics and AI

While Canada was one of the first countries to advocate for creation of a federal AI strategy, there haven’t been similar advances in legislative framework for AI and robotics. Similar to the U.S. and EU, some of the existing Canadian federal and provincial laws can be applied to automated systems. Specifically, Consumer Product Safety Act, Motor Vehicle Safety Act, Privacy Act, and Personal Information Protection and Electronic Documents Act can be of use where user safety, privacy, and data rights are in question [161–164]. The Canadian government has recognized the potential of digital technologies for the services that it provides and can be seen as an active user of AI and robotics. To ensure that these technologies are developed and used ethically, the Treasury Board has released a Policy on Service and Digital to document requirements for privacy, official languages and accessibility, management of service delivery, information management and cyber security in the Canadian government [165]. The policy includes several key directives that dictate the ethical use of AI for government. The Directive on Automated Decision Making addresses the use of AI to make administrative decisions and outlines a process that ensures that AI is being used in a manner that is “compatible with core administrative law principles such as transparency, accountability, legality, and procedural fairness” [166]. One of the key requirements in the Directive is mandatory completion of an Algorithmic Impact Assessment, a questionnaire that is composed of 48 risk and 33 mitigation questions [167]. The questions direct a comprehensive analysis of an AI system, starting from which motivation for the algorithm implementation, and ending with strategies for unbiased data representation. Based on the assessment, an algorithm is assigned an impact level that would inform the stakeholders on the next steps for the algorithm implementation. While the Policy on Service and Digital is currently only used for government projects, it covers ethical principles and practical considerations that are relevant to implementation of any automated system. As such, it could potentially become a general federal policy for AI development and regulation.

In absence of an existing legal framework, companies may resort to signing of detailed contracts with their customers. The contract would then detail company responsibilities, liabilities, and overall serve as a legislative proxy [168]. However, this approach places the responsibility on the consumer to understand the contract and the consequences of signing. The contractual system would not prohibit the company from including unethical clauses, and thus would overall not benefit sustainable and ethical AI or robotics implementation in Canada.

### International policies for ethical robotics and AI

Robotics and AI are relatively new technologies with a great potential; in fact, both belong to a class of technologies that are referred to as “disruptive” for the scale of change they can trigger. Because of that, international organizations have also weighed in on ethical considerations and regulations for AI and robotics. Organizations like the United Nations (UN), the World Bank, and Organization for Economic Co-operation and Development (OECD) have all contributed to the field of AI and robotics by conducting studies and publishing reports that reflect on ethics of these technologies [169–171]. The ethical frameworks and principles identified by the international organizations are of advisory nature and cannot be enforced, but they can serve as a starting point for individual governments to create their own AI and robotics strategies. In other words, there are currently no international policies for ethical AI and robotics. However, international policy might be of the most importance considering the impact and international commercialization of robots and AI. If robots and AI are to be shipped and implemented around the world, a global policy could ensure that they are being used safely and ethically.

While there is no international policy for robotics and AI, there is one particular application of these technologies that might receive international regulation soon. Lethal Autonomous Weapon Systems (LAWS), or so-called “killer robots”, are a cause of active debates held primarily at the UN Convention on Certain Conventional Weapons (CCW) [172]. The purpose of the CCW meetings is to restrict the use of weapons which would cause unjustifiable suffering [173]. During the CCW meetings the member states have acknowledged that LAWS fall under the purview of the International Humanitarian Law but view on the LAWS regulation have been divided. Academics and organizations like the Human Rights Watch have been calling for an international ban on LAWS, but when a preemptive ban on LAWS was proposed at the CCW, it received opposition from countries like Germany, the U.S. and Russia [172, 174, 175]. An outright international ban on LAWS is unlikely to be accepted, so instead a “Killer Robot Treaty” based on aligning views has been proposed [176]. According to the CCW meetings, most States supported the creation of a legally binding regulation for LAWS with specific emphasis on maintenance of human control [176, 177]. Lately the Human Rights Watch has been urging the governments to move from the discussion stage to the policy-writing stage and citing that a number of powerful countries have already been developing LAWS. For countries that are already engaged in LAWS development, the worry is also that their weapons review mechanisms differ greatly, which could lead to production of LAWS whose safety and robustness are not up to standards set out by the CCW [178]. International regulation of LAWS is likely to happen in the near future.

Unfortunately, international regulation of non-military robots does not carry the same sense of urgency. In the absence of international policy, technical standards can be used to establish a baseline level of regulation. The International Organization for Standardization (ISO) is an organization that has published almost 24,000 standards, 40 of which are addressing robotics directly [179]. Some of these standards are quite technical and specific: for example, ISO 15616–4:2008 describes specifications for laser beams used in welding robotics; other standards are based on ethical principles such as safety, equity, and privacy [180, 181]. The latter represent an intriguing opportunity to create industrial regulations based on ethical ISO standards. Without international regulation on ethical robotics and AI, they could be the next best thing to ensure that internationally traded AI and robots don’t compromise users’ rights to safety, equity, or privacy.

## Discussion

This review has largely focused on roboethics and AI policies that were proposed by governments and large international advisory institutions, but other types of organizations such as universities, companies, and non-profits often put forward their own policies for ethical robotics and AI. In fact, while these policies are not binding, they often pioneer ethical concepts which then get implemented in governmental policymaking. Many organizations have published their views on ethical robotics and AI, and the ethical frameworks that emerge from these works are quite similar. A report from the Berkman Klein Center at the Harvard University analyzed 36 documents on ethical AI and has identified eight common themes:

1. Privacy,
2. Accountability,
3. Safety and Security,
4. Transparency and Explainability,
5. Fairness and Non-discrimination,
6. Human Control of Technology,
7. Professional Responsibility, and
8. Promotion of Human Values [182].

Authors have also noted that most recent works on ethical AI have covered all the above themes, which suggests that institutions are aware of key ethical concerns, and that the discourse on the ethics for AI has reached a point of saturation. There are, however, aspects of these themes that have received less coverage in reports on AI principles: the right for data erasure was only covered in 6% of the surveyed works, and only 8% of reports mentioned the ability to opt out of automated decisions [182]. A report by Thilo Hagendorff has also found that issues of certification of AI products and cultural differences for ethical AI systems have received the least attention in the 22 reports that he has surveyed [67]. Altogether, this suggests that the field of ethical AI and robotics is ready to transition from the research stage to the implementation stage. This opinion is further supported by the desire of AI and robotics corporations for guidance on managing of risks that arise from adoption of disruptive technologies [171]. Since the industry is moving faster than the regulators, the International Finance Corporation has developed a Technology Code of Conduct and implementation tools to be used in place of regulatory directives. The Technology Code of Conduct is based on existing ethical frameworks and includes practical steps to ensuring that new technologies support public trust and sustainable innovation [171]. This effort on behalf of the International Finance Corporation demonstrates a trend for technological self-regulation which arose because governments are slow to create their own regulatory standards.

As of 2021, AI and robotics regulation by governmental institutions appears to be at its infancy. Among the regions discussed in this review, Europe has the most rounded approach to AI policies and appears the most prepared to launch full-scale regulation of robotics and AI [4]. The European Commission has committed the most resources to establishment of ethical frameworks and has conducted extensive stakeholder surveys to ensure that proposed regulation is relevant and equitable. The EU has also created a plan for further legislation and a way to address AI and robotics systems based on the risk they carry. Further, EU grants have implemented surveys to ensure that EU-funded technologies are aligned with the developed ethical frameworks for impact assessment. Overall, EU policies for AI and robotics are largely based on public opinions and already contain specific guidance on how the policies are to be implemented.

In North America, the Canadian framework for AI and robotics regulation seems to be more promising. The fact that the Canadian government has already launched laws for regulation of AI within governmental systems signifies its commitment to ethical AI regulation. Additionally, these laws can be expanded to create federal regulation for AI and robotics. The U.S. is currently in the information-collection stage of creating federal AI regulation, which allows for companies like Apple and Facebook to implement high-risk AI systems. Further, there are concerns for whom the U.S. calls “AI experts”- Joichi Ito, who led the MIT media lab and consulted on the ethics of AI, has been revealed to have ties with Jeffrey Epstein, and contributed to Silicon Valley’s efforts to avoid legally enforceable restrictions [66]. For both the U.S. and Canada the existence of case law complicates the regulation of AI and robotics. In cases where decisions are made prior to adoption of federal regulations on AI and robotics, the precedent created could surpass the federal law.

Ultimately, the uptake and implementation of AI and robotics regulation may require research, time, and investment. Both Europe and North America have created ethical frameworks for AI and robotics, and it is now time to transform ethical principles from theory to practice. The following action-steps may be taken by government structures to promote robotics and AI regulation worldwide:

1. Establishment of policies and regulations that enforce roboethics policies;
2. Alignment of funding program’s guidelines with ethical principles;
3. Reform of postsecondary robotics and AI degree programs to include ethical requirements;
4. Establishment of national and international agreements for ethical robotic research, development, and deployment.

The importance of regulation for AI and robotics cannot be overstated: the safety and wellbeing of every member of society depends on responsible use of these disruptive technologies. It is now up to policymakers to ensure that grim predictions of George Orwell and Karel Capek don’t come to life.

## Conclusions

**Fig. 1:**
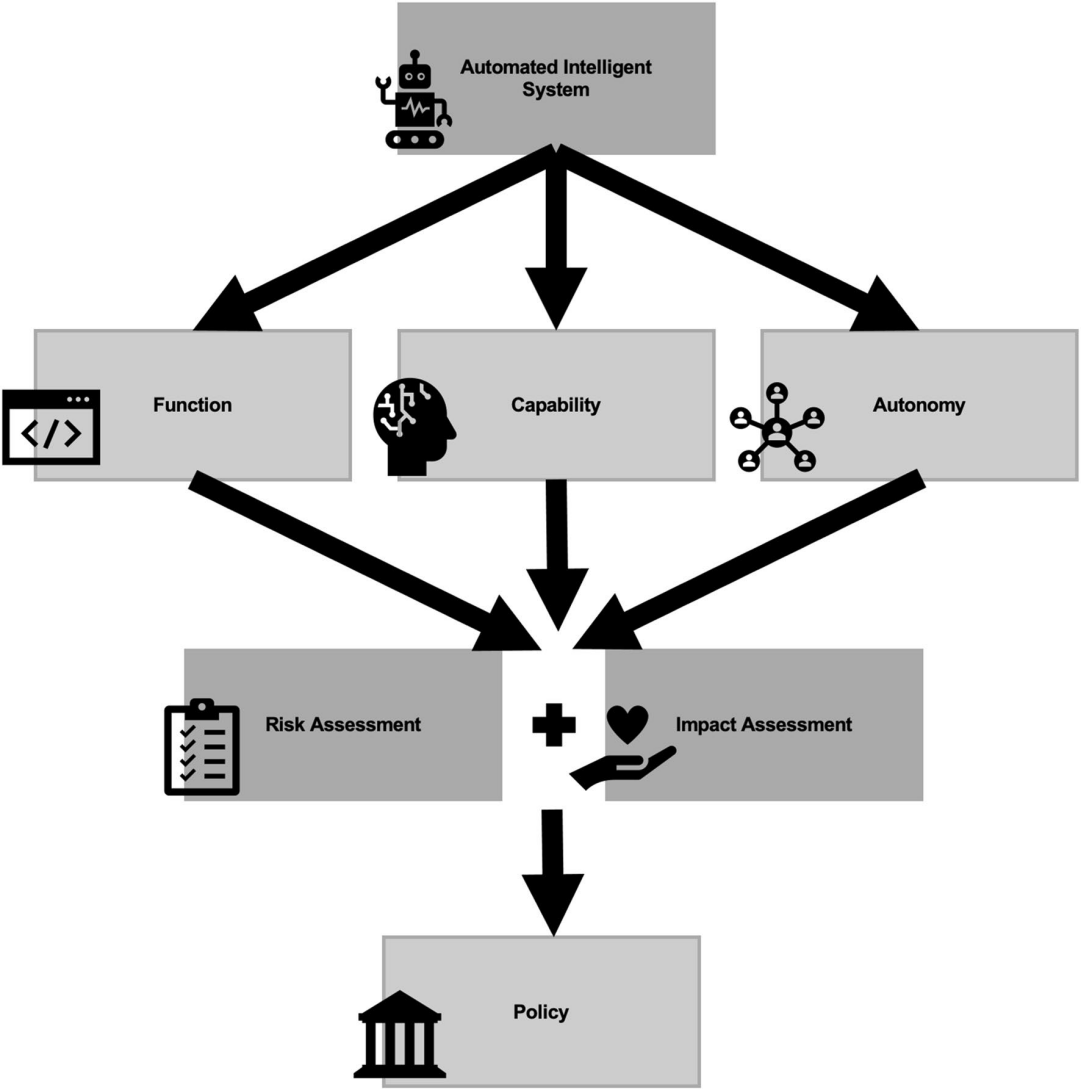
A schematic representing how ethical considerations can become policy by becoming implemented in risk-assessment and impact-assessment tools

Our work summarizes current governmental initiatives in EU, U.S., and Canada to regulate AI and robotics to ensure that new technologies are developed and used in accordance with existing ethical guidelines. In general, policies are built through comprehensive analysis of ethical considerations pertaining to a technology (for example, by considering function, capability, and autonomy categories as outlined in Sect. 3). Governments and organizations have also implemented risk-and impact-assessment tools to facilitate policy adaptation for each robotics case. This process is outlined in Fig. 1. Current ethical frameworks set up by both European and North American governments are being actively translated into enforceable policy, where stakeholders like academic researchers, software engineers, entrepreneurs, and policymakers are collaborating to create a set of regulations that would ensure sustainable innovation and human wellbeing. While neither Europe or North America have a complete regulatory framework for AI and robotics yet, it is likely that that we will see big shifts in regulation of AI-based systems in the next 10 years. The progress of AI policies implemented around the globe can be tracked via the OECD Policy Observatory and through the euRobotics Topics Group "Ethical, Legal and Socio-Economic Issues" directory for policy documents [183, 184].
